# The Effect of Mental Health App Customization on Depressive Symptoms in College Students: Randomized Controlled Trial

**DOI:** 10.2196/39516

**Published:** 2022-08-09

**Authors:** Stephanie G Six, Kaileigh A Byrne, Heba Aly, Maggie W Harris

**Affiliations:** 1 Department of Psychology Clemson University Clemson, SC United States; 2 Department of Computer Science Clemson University Clemson, SC United States

**Keywords:** depression, mental health apps, customization, personalization, cognitive behavioral therapy, avatars, mobile phone

## Abstract

**Background:**

Mental health apps have shown promise in improving mental health symptoms, including depressive symptoms. However, limited research has been aimed at understanding how specific app features and designs can optimize the therapeutic benefits and adherence to such mental health apps.

**Objective:**

The primary purpose of this study is to investigate the effect of avatar customization on depressive symptoms and adherence to use a novel cognitive behavioral therapy (CBT)–based mental health app. The secondary aim is to examine whether specific app features, including journaling, mood tracking, and reminders, affect the usability of the mental health app.

**Methods:**

College students were recruited from a university study recruitment pool website and via flyer advertisements throughout campus. A total of 94 participants completed a randomized controlled trial in which they were randomized to either customization or no customization version of the app. Customization involved personalizing a virtual avatar and a travel vehicle to one’s own preferences and use of one’s name throughout the app. Participants completed a 14-day trial using a novel CBT-based mental health app called AirHeart. Self-report scores for depressive symptoms, anxiety, and stress were measured at baseline and after the intervention. Postintervention survey measures also included usability and avatar identification questionnaires.

**Results:**

Of the 94 enrolled participants, 83 (88%) completed the intervention and postintervention assessments. AirHeart app use significantly reduced symptoms of depression (*P*=.006) from baseline to the end of the 2-week intervention period for all participants, regardless of the customization condition. However, no differences in depressive symptoms (*P*=.17) or adherence (*P*=.80) were observed between the customization (39/83, 47%) and no customization (44/83, 53%) conditions. The frequency of journaling, usefulness of mood tracking, and helpfulness of reminders were not associated with changes in depressive symptoms or adherence (*P*>.05). Exploratory analyses showed that there were 3 moderate positive correlations between avatar identification and depressive symptoms (identification: *r*=−0.312, *P*=.02; connection: *r*=−0.305, *P*=.02; and lack of relatability: *r*=0.338, *P*=.01).

**Conclusions:**

These results indicate that CBT mental health apps, such as AirHeart, have the potential to reduce depressive symptoms over a short intervention period. The randomized controlled trial results demonstrated that customization of app features, such as avatars, does not further reduce depressive symptoms over and above the CBT modules and standard app features, including journal, reminders, and mood tracking. However, further research elucidating the relationship between virtual avatar identification and mental health systems is needed as society becomes increasingly more digitized. These findings have potential implications for improving the optimization of mental health app designs.

**Trial Registration:**

Open Science Framework t28gm; https://osf.io/t28gm

## Introduction

### Background

Ranked as the 6th most expensive health condition, depression costs the United States approximately 326.2 billion dollars in treatment and workplace costs in 2018 [1]. Depression is highly prevalent across all age groups, genders, and racial groups, causing anhedonia, irritability, extreme sadness, and other emotional and physical symptoms [2]. This rise in spending parallels the rate of diagnosis of major depressive disorder, which has increased 7-fold over the past 5 years [3]. Certain therapeutic techniques, including cognitive behavioral therapy (CBT), have been implemented to alleviate depressive symptoms.

CBT can be efficacious in reducing depressive symptoms and improving the quality of life in both clinical [4-6] and nonclinical populations [7] of multiple age groups [8,9]. In addition to depression, CBT has been successful in treating symptoms of anxiety and stress [10-15]. Depression is a complex mental health condition that is often comorbid with anxiety and exacerbated by stress [16-18]. Therefore, reducing the symptoms of depression may further aid in alleviating the negative manifestations present in other comorbid or associative disorders.

CBT has been shown to be effective in reducing symptoms of depression not only in in-person therapy environments but also in mobile apps [19,20]. The combination of CBT and mobile technology has burgeoned in the last 10 years, with an estimated 10,000 to 20,000 mental health apps currently existing in the Apple App Store and Google Play Store [21]. Despite its prevalence, there is a dearth of research investigating the interaction between mobile CBT and technological features, such as mobile journaling, reminders, mood tracking, and customization, on mental health symptom reduction. Certain features may complement, augment, or detract from CBT delivery and its effectiveness in symptom reduction. Thus, this study sought to experimentally address this gap in knowledge by investigating how specific app features, including the use of customized avatars, would affect depressive symptoms and adherence.

Computerized CBT (cCBT) is a web-based form of CBT that is accessed through a computer, smartphone, or tablet [22]. Many cCBT mental health apps, such as Space from Depression and MoodGYM, use a time line similar to brief CBT, which is typically 4 to 8 sessions or modules [23-25]. This type of CBT has shown effectiveness in reducing depressive symptoms in both clinical [26,27] and subclinical [28,29] depressive populations of varying ages, although the results from 2 meta-analyses suggest that cCBT mental health apps may be more effective for subclinical than for clinical levels of depression [30,31]. One caveat of this type of therapy is its low adherence rate [32,33]. A proposed explanation for the low adherence rates includes individuals not progressing as quickly as expected, leading to the conclusion that treatment is not effective [34]. In addition, participants also reported quitting if they had negative expectations about their treatment outcomes. Individuals who do not believe that their treatment will be effective often perceive it as inconvenient and quit trying another therapy technique or spending their time elsewhere [35]. Overall, adherence remains a problem in a variety of cCBT programs, but various elements and tools can be used to encourage adherence.

Researchers from different disciplines have presented multiple suggestions to counteract the low adherence rates. One notable suggestion is gamification, which can be defined as the implementation of game elements, such as challenges, rewards, badges, or levels, into a system [36,37]. Gamification has emerged as one of the most widely used solutions for increasing adherence [38,39]. Although it may not provide any additional benefits in reducing depressive symptoms when coupled with therapeutic techniques such as cCBT [40], it has been shown to increase adherence in therapy trials for a variety of mental health disorders, such as depression and anxiety [41,42]. Some specific technological elements, including journaling, mood tracking, and reminders, have been shown to effectively increase engagement *and* aid in the reduction of depressive symptoms in observational studies [43-46]. However, few studies have experimentally investigated the effect of these elements on adherence and mental health symptoms within mental health apps.

One technological feature that has been largely overlooked in mental health app research is customization. In the technology domain, customization is the process of changing a product or interface to make it more personalized to an individual’s preferences or needs. Customization permeates through mobile technology, such as the Apple iPhone, which allows the user to create custom alarms, reminders, or ringtones. More specifically, customization within mobile apps can include the creation of a self-representative avatar. An avatar is a virtual representation of a genuine user, where the user can alter various features, such as hairstyle, clothes, skin color, and facial features. Users may have a strong preference for programs that include customizable avatars. For example, a qualitative study conducted focus groups and interviews with adolescents exhibiting depressive symptoms to investigate the usability of a cCBT fantasy game with avatars (SPARX) [47]. This study found that participants enjoyed the option of personalizing their characters, because they could easily relate to the personalized characters [47]. On the basis of prior research, this study used avatar customization to increase app adherence and identification with the avatar.

To the best of our knowledge, only one study has experimentally examined the connection between customization and mobile mental health interventions [48]. Participants were randomized to a condition in which they either created their avatar or were assigned a random avatar that they could not personalize. Baseline anxiety levels were assessed, and participants completed either an attention bias modification training or a no training control activity. Ultimately, the study found that the participants’ ability to customize their avatar increased their resilience to the induction of negative moods, their identification with the avatar, their engagement, and the efficacy of therapeutic training [48]. This study was among the first to directly investigate the relationship between customization and anxiety, as well as how avatar customization affects identification, engagement, and efficacy. However, it is unclear whether customizable avatars can affect *depressive* symptoms. Consequently, this study sought to fill this gap in prior research by investigating the efficacy of avatar customization within a CBT mental health app for depressive symptoms.

### This Study and Hypotheses

#### Overview

Overall, prior research suggests that customization has the potential to influence mental health symptoms, such as anxiety, and increase adherence within a mental health app. When combined with CBT, customization can increase adherence by augmenting self-representation and escapism. The addition of reminders and mood tracking may further increase adherence by promoting app engagement and use of therapeutic tools. The primary aim (aim 1) of this study was to examine the effect of customization on depressive symptoms and adherence to a mental health app, controlling for depression diagnosis. We controlled for depression diagnosis, because prior research has shown that cCBT mental health apps may be more effective for those with subclinical compared with clinical levels of depression [30,31]. The secondary aim (aim 2) was to explore how the perceived usability of the mental health app and its internal features (mood tracking, reminders, and journaling) influenced depressive symptoms and adherence. To examine these aims, this study used a 2-arm randomized controlled trial (1:1 allocation ratio) to compare a mental health app with customization (the intervention) and without customization (the active control) on adherence and depressive symptoms. The specific hypotheses of this study are outlined as follows.

#### Primary Hypotheses for Aim 1

The primary hypotheses for aim 1 include the following:

- H1: It is hypothesized that the ability to customize a personal avatar will further reduce depressive symptoms compared with the active control.
- H2: It is hypothesized that the level of adherence, measured through the completion of the log-in questionnaires, will be higher in the customization condition than in the active control condition.

#### Primary Hypotheses for Aim 2

The primary hypotheses for aim 2 include the following:

- H3: It is hypothesized that as the number of journal entries increases, symptoms of depression will decrease, and adherence level will increase.
- H4: It is hypothesized that higher usefulness scores for the reminders will lead to a higher level of adherence and decreased depressive symptoms.
- H5: It is hypothesized that greater use and understanding of mood patterns will lead to a higher level of adherence and decreased depressive symptoms.

#### Exploratory Aims and Hypotheses

It is possible that individuals with depressive symptoms may differ in their identification with their avatar; however, there is limited prior research aimed at examining this possible relationship. Thus, as an exploratory analysis, we examined whether depressive symptoms were associated with differences in identification with one’s avatar. In addition to investigating the effect of CBT-based mental health app features on depression symptoms, this study also examined the effect of customization on anxiety and stress symptoms. Customization is expected to reduce anxiety and stress.

## Methods

### Participants

The target population for this study was college students enrolled full time. To address H1, an a priori power analysis (*F*-test, repeated measures ANOVA, and within-between interaction) was performed using the G*Power 3.1 (Universität Kiel). The analysis sought to determine the number of participants necessary to maintain a power level of 80% to detect a possible effect at the *P* value of .05 level with 2 groups and 2 measurement time points. A meta-analysis was conducted to determine whether various gamification elements improved the reduction of depressive symptoms in different mental health apps [40]. Cohen *f* for this experiment (*f*=0.16) was calculated from the Hedges *g* (*g*=0.32) provided in the meta-analysis, because both projects investigated mental health apps for depression. According to this analysis, a sample of 80 participants would be needed to have 80% power to detect an effect. Multimedia Appendix 1 presents the log of this power analysis.

**Figure 1:**
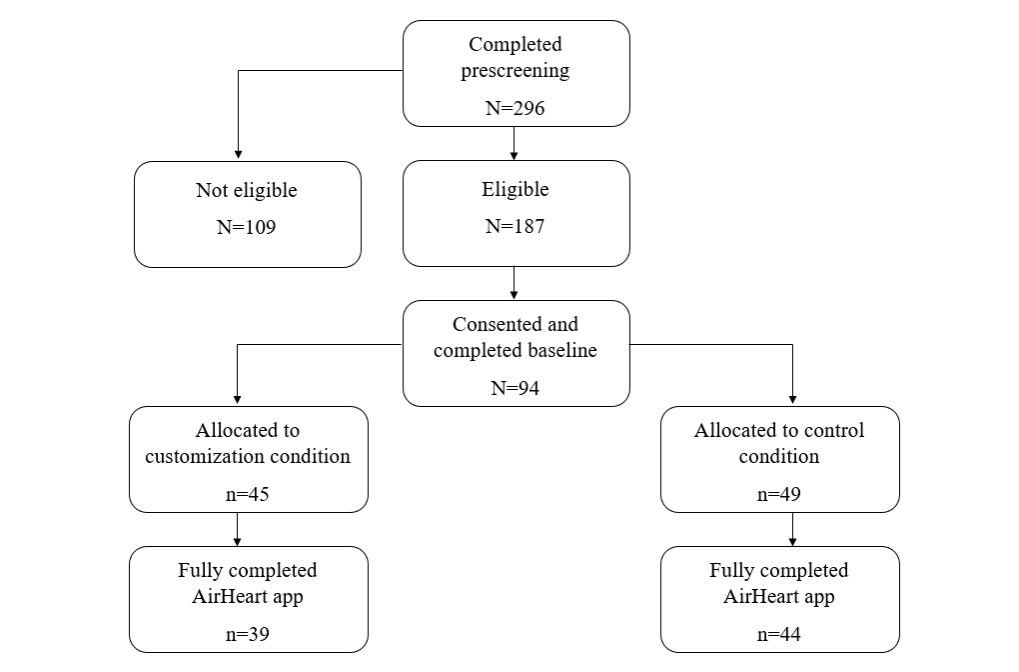
Participant flow diagram.

To recruit a sample representative of the depressive population, 296 participants were screened before beginning the study. Participants were recruited through SONA Systems software, a cloud-based participant recruitment pool or flyer advertisements. The recruitment flyer and web-based information said only that “beta testers” were needed, and compensation would be provided. Participants received a course or extra credit for their classes, if applicable, and a US $20 Amazon gift card if they fully completed the study. Despite the large prescreening sample size, many individuals (n=109) were ineligible or declined to complete the study (n=94). A total of 94 students from Clemson University completed the prescreening and preassessment, and 88% (83/94) of students completed all 3 mandatory requirements (the prescreening, preassessment, and postassessment questionnaires). Participants were randomly assigned by the computer database to either the customization intervention (39/83, 47%; mean 20.462, SD 2.437) or the active control group (44/83, 53%; mean 20.978, SD 2.633). The recruitment, screening, and study period lasted from February 2022 to May 2022, when the semester was concluded. Figure 1 shows visualization of the participant flow diagram. Multimedia Appendix 2 presents the inclusion and exclusion criteria.

### Materials

#### AirHeart App

AirHeart is a CBT mental health app designed exclusively for this study to aid in reducing depressive symptoms. This app immerses participants into a world of discovery as they travel in a hot-air balloon to the 7 wonders of the modern world. Each stop along their journey provides new depressive symptom management techniques in the form of 7 cognitive behavioral training modules, which encourage the implementation of new cognitive strategies and offer new healthier behaviors. The scripts for each module are presented in Multimedia Appendix 3. AirHeart includes other features, such as log-in questionnaires, mood tracking, journaling, and reminders. Further details of these features are provided in Multimedia Appendix 4.

#### Experimental Manipulation: Customization and Personalization

A total of 2 different elements within the intervention group were customized: an avatar and a hot-air balloon. One of the first steps of the AirHeart tutorial, led by both the app and a research assistant introducing the participants to the app, was avatar customization. This customization prompted the participants to create an avatar to embody themselves by tailoring the avatar’s skin, eye, hair color, and clothes. The participants also customized their avatar’s hot-air balloon, specifically tailoring the color of the balloon. Multimedia Appendix 5 provides customization instructions, avatar examples, and hot-air balloon examples.

In addition to the customization of the avatar and hot-air balloon, the app asked for the participant’s name to personalize the journal and mood tracking chart to the user. Use of the participant’s name could be observed on the cover of the journal and as a header on the mood tracker page.

#### Active Control Group: No Customization or Personalization

The control version of AirHeart did not include avatar or hot-air balloon customization. The control avatar was designed as a gray, gender-neutral person with no specific features, and the hot-air balloon was gray colored. This version of AirHeart asked the participant’s name but did not use it in the journal or mood tracker. The avatar for the active control condition is presented in Multimedia Appendix 6.

### Study Design

This study used a 2 (app condition: customization vs no customization)×2 (time: baseline vs 14-day postintervention period) mixed design, controlling for depression diagnosis (coded as major depression disorder [MDD]: yes vs no). The app condition followed a between-subjects design, time followed a within-subjects design, and depression diagnosis represented a covariate. Participants completed a baseline training and setup session along with baseline questionnaires via a face-to-face assessment; thereafter, participants completed the intervention and postintervention questionnaires on the web.

### Measures: Mental Health Symptoms

All questionnaires were based on self-reported data from participants during the past 2 weeks.

#### Depressive Symptom Questionnaire

The Patient Health Questionnaire (PHQ; PHQ-8) was administered at baseline and after the intervention, and the PHQ-4 was administered as part of the app design. Compared with the PHQ-9, the PHQ-8 omitted questions regarding self-harm and suicidal thoughts. The AirHeart app was not specifically designed to alleviate suicidal ideologies, and it falls out of the scope of this study. The PHQ-8 is an 8-item questionnaire that assesses the frequency of depressive symptoms over the past 2 weeks on a Likert scale, ranging from 0 (*not at all*) to 3 (*nearly every day)*. The PHQ-8 indicates symptoms of minimal depression (score of 0-4) to severe depression (score of 17-24) [49]. The PHQ-4 is a 4-item, shortened version of the PHQ-8.

#### Anxiety Symptom Questionnaire

The Generalized Anxiety Disorder-7 (GAD-7) is a 7-item questionnaire measuring anxiety over the past 2 weeks, ranging from minimal anxiety (0-4) to severe anxiety (15-21). Participants responded to the 7 questions on a 4-point Likert scale, ranging from 0 (*not at all*) to 3 (*nearly every day*). This questionnaire asks about the frequency of nervousness, worry, relaxation, irritability, restlessness, and fear [50].

#### Stress Questionnaire

The Perceived Stress Scale (PSS; PSS-10) was administered at baseline and after the intervention, and the shortened PSS-4 was administered as part of the app design. The PSS is a 10-item questionnaire measure of stress over the past month, with scores ranging from 0 to 40, with 0 to 13 indicating low stress and 27 to 40 indicating high stress [51]. This questionnaire, which is a shortened version of the PSS-14, presents scenarios to the user and asks them to rank their responses on a 5-point Likert scale, ranging from 0 (*never*) to 4 (*very often*).

#### Positive and Negative Affect Scale—Short Form

The Positive and Negative Affect Scale—Short Form is a 20-item questionnaire investigating participants’ positive and negative emotions. This questionnaire contains 2 scales, one for positive affect and the other for negative affect, with both scores cumulatively ranging from 20 to 100 or 10 to 50 for each scale. Some examples of the adjectives on the positive affect scale used to describe participants’ current feelings include “interested,” “strong,” or “excited,” whereas the negative affect scale includes adjectives such as “distressed,” “hostile,” or “scared” [52].

### Measures: Usability and Adherence

#### Demographic Questionnaire

Various demographic factors, such as the participant’s gender and age, were recorded during both the pre- and postintervention surveys. In addition, during the preintervention period, questions regarding the participants’ prior mental health app use and clinical diagnosis were included.

#### Usability Questionnaire

The System Usability Scale (SUS) is a 10-item questionnaire measuring the usability of a system or product using a 5-item Likert scale, ranging from “strongly agree” to “strongly disagree.” The scale ranges from 0 to 100, with any score of ≥68 being average and any score <68 is below average [53,54].

In addition to the SUS, the postassessment questionnaire also collected self-report data regarding efficacy, convenience, and usefulness of the reminders, mood tracker, journal, positive feedback, and storytelling elements and whether participants would continue to use the app if it was available for longer than 14 days.

#### Adherence Data

Adherence data were collected each day the participants logged in to the app. The number of log-ins along with the days when the participants used the app were compiled.

### Procedure

#### Ethics Approval

Approval was first received from the Clemson University Institutional Review Board (IRB2021-0879). In addition, this study was preregistered on the open science framework [55].

#### Screening

Before participants began the baseline session using the AirHeart app, they completed the PHQ-8 screening. To be eligible for the study, participants received a score of ≥5, indicating at least mild symptoms of depression. The participants had access to the PHQ-8 via a Qualtrics survey. If a participant received a score of ≤4, they were thanked for their time but were not provided with the passcode to join the full AirHeart study.

#### Baseline Session

At the initial in-person baseline session, participants provided written informed consent and completed the PHQ-8, GAD-7, PSS-10, demographic survey, and filler questions on campus laboratory computers via the Qualtrics web platform. The informed consent document included privacy statements (eg, participants’ email would be retained) and campus mental health hotline information. Participants were then randomly assigned to either the intervention or the active control condition. Participants were granted access to their version of the app on their mobile phones. The participants were blinded to their condition and were thus unaware of the experimental manipulation. Blinding of the researcher to the condition was impossible because the researchers were required to assist participants in the download procedure of the AirHeart app. A physical step-by-step guide was given to the participants, who were instructed to follow along with the guide and were encouraged to ask questions along the way.

To begin, the participants opened the app, which asked them to register for an account, after which they met their avatars. In the intervention condition, participants customized their avatar to look like themselves, and the active control was simply given the gray avatar. Next, participants completed the 16-item log-in questionnaire consisting of the PHQ-4, PSS-4, and 8 positive affect questions from the Positive and Negative Affect Scale—Short Form and their first journal entry. After finishing the prerequisites for the CBT modules, the home page was shown, and participants were encouraged to start exploring their first wonder (and thus the first CBT module). Once the first CBT module, “wonder,” was finished, participants were prompted to set up reminders on their phone. They were then informed that US $20 compensation would be given if they completed the 7 modules, 7 journal entries, 7 log-in questionnaires, and a postintervention survey. Once the participants completed the steps on the guide in the app, they exited the laboratory.

#### Intervention Period

After the initial session, participants completed 6 more modules over the following 2-week period that were nearly identical to the app experience in the baseline session. Multimedia Appendix 7 provides a detailed outline of the intervention period.

#### End-of-Intervention Assessment

A survey was emailed to the participants 1 day after the completion of the 2-week intervention period. This survey contained the original 3 questionnaires, the PHQ-8, GAD-7, and PSS-10 as well as a usability scale (SUS) and questions regarding the relationship of the participant to the avatar, identification, and usability elements of the app features.

### Data Analysis

To test aim 1, we performed a 2 (intervention type: customization vs no customization control condition)×2 (time: baseline vs postintervention) mixed effects analysis of covariance, controlling for depression diagnosis. This analysis was conducted to identify the effect of customization on depressive symptoms over time (H1). We also conducted a 2-tailed independent sample *t* test for the adherence outcome, the number of log-ins (H2). All analyses were performed using an intent-to-treat approach.

To test aim 2, a correlation analysis was performed between depressive symptom scores and journal entries (H3). Multiple linear regressions were performed to assess the effect of reminder usefulness (H4) and mood tracking understanding and usefulness (H5) on adherence and depressive symptoms.

## Results

### Participant Characteristics

A total of 296 participants completed the prescreening, and 187 (63.2%) qualified for the full AirHeart study, but only 94 consented and completed the preassessment questionnaire. A total of 83 participants also completed the postassessment questionnaire (mean_age_ 20.771, SD_age_ 2.539 years); 33% (13/39) reported a diagnosis of MDD in the intervention (mean_age_ 20.487, SD_age_ 2.516 years), and 25% (11/44) reported MDD in the control condition (mean_age_ 21.091*,* SD_age_ 2.701 years). In addition, 28% (11/39) of participants reported prior use of a mental health app in the intervention condition, and 23% (10/44) of the participants reported prior use of a mental health app in the control condition. Further information regarding baseline participant characteristics is presented in Table 1.

**Table 1:** Baseline participant characteristics overall and by condition.

Variables		Overall sample (N=83)	Customization condition (n=39)	No customization condition (n=44)	Significance level (*P* value)	
Age (years), mean (SD)		20.77 (2.54)	20.46 (2.44)	21.05 (2.62)	.30	
**Gender^a^**						.37
	Female, n (%)	60 (72)	28 (72)	32 (73)		
	Male, n (%)	19 (23)	8 (21)	11 (25)		
	Nonbinary, n (%)	4 (5)	3 (7)	1 (2)		
Prior app use (yes), n (%)		21 (25)	11 (28)	10 (23)	.57	
Major depression disorder diagnosis (yes), n (%)		24 (29)	13 (33)	11 (25)	.41	
Depression scores, mean (SD)		9.39 (4.99)	9.00 (4.82)	9.73 (5.16)	.51	
Anxiety scores, mean (SD)		8.60 (4.52)	7.62 (4.05)	9.48 (4.78)	.06	
Stress scores, mean (SD)		22.39 (3.91)	22.56 (3.73)	22.23 (4.10)	.70	

^a^*F*_1,81_=0.82.

### Descriptive Information for System Usability

The SUS reached an average of 54.349 (SD 18.293; range 25-85) on a scale from 0 to 100, which was below the average of 68. This below average score indicates that the AirHeart app is below the average usability point, suggesting the necessity of an update to make the app potentially less complex, cumbersome, and more intuitive; however, the large SD indicates a wide range of differing opinions.

### Aim 1 Analyses

#### Effect of Customization Versus No Customization on Depressive Symptoms

In accordance with the first hypothesis (H1), a 2 (time: baseline and 14-day postassessment period) ×2 (condition: customization or control) mixed analysis of covariance, controlling for depression diagnosis (MDD: *yes vs no*), was conducted to investigate whether the customization of a virtual avatar would further reduce depressive symptoms over time. A significant main effect of time was observed (*F*_1,79_=8.044; *P*=.006; η_p_^2^=0.092); however, no other effects demonstrated significant differences: time×condition (*F*_1,79_=1.965; *P*=.17; η_p_^2^=0.024); time×diagnosis (*F*_1,79_=2.575; *P*=.11; η_p_^2^=0.032); time×condition×diagnosis (*F*_1,79_=1.269; *P*=.26; η_p_^2^=0.016); condition×diagnosis (*F*_1,79_=.026; *P*=.87; η_p_^2^<0.001). Thus, the app was effective in reducing depressive symptoms across all participants, but customization had no significant effect. Thus, H1 was not supported.

#### Effect of Customization Versus No Customization on App Adherence

The independent sample *t* test results showed no significant difference between the customization and no customization active control conditions on AirHeart app number of log-ins (*P*=.95). These results do not support H2. The descriptive statistics for the log-in questionnaire, modules, and journal entries are presented in Table 2.

**Table 2:** Descriptives for log-in questionnaires, journal entries, and modules completed overall and by condition.

Variables	Overall sample (N=83), mean (SD)	Customization condition (n=39), mean (SD)	No customization condition (n=44), mean (SD)	Significance level (*P* value)
Log-in questionnaire	7.49 (2.42)	0.51 (2.16)	0.48 (2.65)	.95
Journal entries	6.28 (2.45)	6.13 (1.77)	6.41 (2.94)	.61
Completed modules	6.75 (0.84)	6.92 (0.35)	6.59 (1.09)	.07

### Aim 2 Analyses

#### Relationship Between Depressive Symptoms and App Features

Journal entry frequency was not significantly associated with changes in depressive symptoms (postintervention minus baseline depressive symptom levels: *r=*−0.076; *P*=.49).

A multiple linear regression with the 6 reminder statements predicting changes in depressive symptoms did not reach statistical significance (*F*_6,68_=1.305; *P*=.27), nor were any of the individual 6 statements associated with depressive symptoms (*P*=.08*-*.88). Table 3 presents the regression results.

A similar regression with the 4 mood tracking and usefulness questions (Table 4) predicting changes in depressive symptoms also failed to reach significance for the overall model (*F*_4,68_=0.339; *P*=.85) and individual items (*P*=.32-.99). These results failed to support H3 to H5 for the depressive symptom outcome measure.

**Table 3:** Results of the multiple linear regression between reminder variables and change in depressive symptoms^a^.

Reminder variables	ß	*t* test (*df*)	Significance level (*P* value)
The reminders helped me to remember to complete my modules	−.057	−0.382 (68)	.70
The reminders were annoying	−.338	−1.792 (68)	.08
The reminders were inconvenient	.283	1.206 (68)	.23
I would turn off the reminders if I could	.296	1.377 (68)	.17
The reminders helped improve the quality of the app	.200	1.214 (68)	.23
I got excited when I saw the reminders	.021	0.151 (68)	.88

^a^Changes in depressive symptoms were defined as postintervention scores minus baseline depressive symptom scores.

**Table 4:** Results of the multiple linear regression between mood tracking variables and change in depressive symptoms^a^.

Mood tracking variables	ß	*t* test (*df*)	Significance level (*P* value)
The mood tracking helped me understand my pattern of moods	−.159	−0.997 (68)	.32
I liked being able to track my mood and symptoms	.105	0.584 (68)	.56
I did not use the mood tracking	−.002	−0.016 (68)	.99
The mood tracking made me want to use the app	−.067	−0.448 (68)	.66

^a^Changes in depressive symptoms were defined as postintervention scores minus baseline depressive symptom scores.

#### Relationship Between Adherence and App Features

In terms of adherence, journal entry frequency was positively associated with adherence (*r*=0.638; *P*<.001). In addition, the overall model for multiple linear regression with the 6 reminder statements predicting the number of log-in questionnaires did not reach statistical significance (*F*_6,68_=0.390; *P*=.88). None of the individual reminder statement predictors were significant (Table 5) in predicting usefulness on their own; therefore, H4 was not supported.

In addition to journaling and reminders, questions regarding the use and understanding of mood trackers were investigated through multiple linear regression. The overall model did not reach significance (*F*_4,68_=2.115; *P*=.09), nor did any individual factor reach the level of significance. Therefore, H5 is not supported. Table 6 presents the regression results.

**Table 5:** Results of the multiple linear regression between reminder variables and the number of log-in questionnaires completed.

Reminder variables	ß	*t* test (*df*)	Significance level (*P* value)
The reminders helped me to remember to complete my modules	.097	0.629 (68)	.53
The reminders were annoying	.115	0.587 (68)	.56
The reminders were inconvenient	−.016	−0.065 (68)	.95
I would turn off the reminders if I could	−.049	−0.219 (68)	.83
The reminders helped improve the quality of the app	−.114	−0.660 (68)	.51
I got excited when I saw the reminders	.156	1.089 (68)	.28

**Table 6:** Results of the multiple linear regression between mood tracking variables and the number of log-in questionnaires completed.

Mood tracking variables	ß	*t* test (*df*)	Significance level (*P* value)
The mood tracking helped me understand my pattern of moods	.230	1.516 (68)	.13
I liked being able to track my mood and symptoms	−.077	−0.455 (68)	.65
I did not use the mood tracking	−.238	−1.814 (68)	.07
The mood tracking made me want to use the app	−.084	−0.588 (68)	.56

### Exploratory Analyses

#### Effect of Customization Versus No Customization on Anxiety and Stress Symptoms

A 2 (time: baseline and 14-day postassessment period)×2 (condition: customization or control) mixed ANOVA was conducted to investigate whether the customization of a virtual avatar would further reduce anxiety symptoms over time. The effects of time (*P*=.08), condition (*P*=.13), and time×condition interaction (*P*=.28) were all nonsignificant. Customization did not influence anxiety symptoms, and the AirHeart app did not significantly reduce anxiety symptoms from the baseline.

Mixed ANOVA results for stress indicated that stress levels significantly declined from baseline to after the intervention (*F*_1,79_=11.438; *P*=.001; η_p_^2^=0.126), but customization did not influence stress symptoms (time×condition: *P*=.29). The main effect of the condition (*P*=.21) was nonsignificant.

#### Relationship Between Depressive Symptoms and Avatar Identification

Bivariate correlations were conducted to investigate the relationship between depressive symptoms and identification with one’s avatar. Significant associations were observed between depressive symptom scores and the statements “I identified with my avatar” (*r*=−0.312; *P*=.02), “I felt a connection with my avatar” (*r*=−0.305; *P*=.02), and “my avatar was not like me” (*r*=0.338; *P*=.01); however, no other statement reached significance. The results for the depressive symptoms are presented in Table 7. Multimedia Appendix 8 presents the results for anxiety and stress symptoms.

**Table 7:** Correlations between Patient Health Questionnaire-8 scores after 14 days and the Avatar Identification Questionnaire.

Avatar Identification Questionnaire	Pearson correlation	Significance level (*P* value)
Identified with avatar	−0.312^a^	.02
Connection with avatar	−0.305^a^	.02
Avatar was not like me	0.338^a^	.01
Avatar is more accomplished	0.174	.20
I like my avatar	−0.169	.21
Avatar made AirHeart more enjoyable	−0.217	.11
Avatar made me want to use AirHeart	−0.244	.07
Avatar helped during modules	−0.189	.16

^a^Correlation is significant at the .05 level (2-tailed).

## Discussion

### Principal Findings

This study tested the effectiveness of customization within a novel CBT-based mental health app (AirHeart) on depressive symptoms and app adherence. Customization focused on virtual avatar self-representations, vehicle representations, and the use of an individual’s name throughout the app. The results indicated that, on average, depressive symptoms decreased over time in all participants. However, customization and personalization of app features did not lead to a further reduction in depressive symptoms (H1) or adherence (H2) compared with the control group. Therefore, although depressive symptoms declined overall, customization and personalization did not exert a significant benefit on symptom reduction or adherence. Instead, the core features of cCBT implemented within an app appear to be independently effective.

The finding that depressive symptoms declined from baseline after the 14-day app intervention period is consistent with randomized controlled trials evaluating cCBT mental health app efficacy [20,23,56-59]. More specifically, this finding aligns with other studies showing that brief CBT-based mental health apps, which consist of a short 4- to 8-module intervention, can mitigate symptoms of depression [23]. However, this study did not include a no app control group. Although the results showed a significant reduction in symptoms from baseline to after the intervention, the primary goal of the study was to compare customization within 2 variants of a CBT-based mental health app rather than to compare the effectiveness of the AirHeart app to a control group.

In addition to depressive symptoms, we explored the effect of AirHeart and AirHeart feature customization on anxiety and stress symptoms. The results indicated that self-reported stress levels decreased from baseline to after the intervention, but there were no significant changes in anxiety symptoms. Similar to the results for depressive symptoms, customization did not significantly affect stress or anxiety levels. The AirHeart cCBT modules targeted strategies for alleviating depressive symptoms but were not designed for anxiety or stress symptoms. Thus, the reduction in stress levels may represent an added benefit of the cCBT mental health app.

The second aim of this study was to examine whether specific app features, including journaling, mood tracking, and reminders, were related to depressive symptoms and adherence. Journal entry frequency was linked to adherence but not to depressive symptoms. Users who journaled more frequently had higher adherence levels. However, given the correlative nature of this result, the directionality of this relationship is inconclusive. It is possible that individuals who used the app more frequently were also incidentally journaling more. Furthermore, in contrast to our hypotheses, the results showed that the likability and usefulness of mood tracking and the helpfulness of reminders did not impact depressive symptoms or adherence. This finding was surprising, given that prior qualitative, mixed methods, and review studies have shown evidence that journaling, mood tracking, and reminder features within mental health apps for depression can effectively increase engagement and aid in the reduction of depressive symptoms [44-46]. We speculate that the nature of CBT module delivery—exploring depressive symptom management strategies by navigating through the wonders of the world—may have been more engaging than the other features that we assessed. Future research is needed to understand the role of these specific app features in mental health symptoms and adherence.

Despite the prevalence of virtual avatars in apps, video games, and virtual meeting platforms, there is an exceptionally limited work characterizing individual difference factors that influence the connection with a self-representative virtual avatar. As an exploratory analysis, we examined the relationship between virtual avatar identification and depressive symptoms. An interesting finding of this study was that individuals with higher levels of depressive symptoms did not identify or connect with their virtual avatars. This finding was observed across both the customization and no customization control conditions. Thus, this negative relationship between depressive symptoms and avatar identification was observed, regardless of the level of customization and personalization within the app. This finding diverges from other research, suggesting that participants would form an attachment and identify with their virtual avatar [48,60-62]. Although this finding may not support our hypothesis, it suggests that individuals with higher depression symptoms may have more difficulty in identifying with virtual self-representative avatars, regardless of aesthetics or similarity to themselves. This finding may be explained by specific symptoms of depression. In particular, depressed individuals often experience increased levels of self-loathing [2], which could reduce their feelings of positive connection with a self-representative avatar. In other words, if an individual does not like themselves, it is reasonable to expect that they would not like a virtual representation of themselves. Alternatively, it is possible that the lack of certain avatar customization options within the AirHeart app that are present within other apps may have reduced identification.

With highly customizable avatars common in popular video games, such as The Sims, Skyrim, and Animal Crossing, as well as on mobile devices, such as Bitmojis, both unconscious requirements and conscious judgments for not providing specific hairstyles, skin color, accessories, or clothing may reduce the identity of one’s avatar. Although several studies have examined player expectations for in-game behavior, little research has been documented regarding players’ expectations for the game avatar creation system [63,64], and in particular, how mental health conditions, such as depression, may influence avatar use and identification.

### Limitations

This study has certain limitations. First, technical difficulties within the AirHeart app impacted the experiences of a few participants. Participants who at times lacked a steady Wi-Fi connection reported having difficulty accessing the log-in questionnaires at the correct time. Although all participants who reached out with difficulties responded within a 24-hour period either by the researcher (SGS) or developer (HA), this could have impacted the usability and effectiveness of the AirHeart app. Furthermore, this limitation may have affected the SUS scores reported by the participants.

In addition, owing to a technical error with the Qualtrics website along with user error, one questionnaire regarding the participants’ opinions about the features within the AirHeart app was not saved to the Qualtrics survey; thus, participants did not originally complete this questionnaire in their postassessment period. Although a total of 16 participants completed the questionnaire as a separate survey, there was a 19% (16/83) participant loss for this questionnaire due to this error. This loss of data impacted H3 and H4, which stated that the number of journal entries and usefulness of reminders would both be associated with heightened adherence and diminished depressive symptoms.

Finally, customization options were limited to skin, eye, hair color, and clothing choice. Participants were not able to customize their facial expressions, facial features (eg, facial hair and piercings), or personal features such as tattoos. In addition, customization lacked some level of religious and accessibility inclusivity, including the lack of wheelchair, hijab, or yamaka options. Individuals may feel that such features are a strong reflection of their identity, and without such options, they may feel detached from their avatar.

### Future Directions

The AirHeart mental health app provides a foundation for future mental health app development and informs work on avatar development. Future studies could replicate this design with improved graphics and more customization options for both the avatar and hot-air balloons. Animation could be added as a way of increasing the potential connectivity with one’s avatar. More research is needed on the overall connection with human avatars, specifically on whether people identify more with avatars that look like them or potentially look different, whether through age, gender, or even species. Future studies are needed to characterize the guidelines and requirements for high-level avatar identification. In addition, ways to increase such avatar identification for individuals with mental health conditions, such as depression, are needed. Finally, future work is needed to identify whether these findings can be generalized to a broader, nonstudent population.

### Conclusions

Numerous mental health apps use avatars, customization, and gamification; however, the therapeutic benefits of these features appear limited [40]. The findings of this study may provide insights into the usability and functionality of certain mental health app features to further refine app development. This study provides empirical evidence that customization features within cCBT mental health do not provide significant benefits for symptom reduction or adherence over and above the CBT intervention itself. Identifying specific app features that improve app effectiveness is required to optimize the design of mental health apps and improve users’ mental health symptoms.

## Appendix

Multimedia Appendix 1 Log of the study power analysis.

Multimedia Appendix 2 Inclusion and exclusion criteria.

Multimedia Appendix 3 AirHeart app cognitive behavioral therapy module scripts.

Multimedia Appendix 4 AirHeart app features.

Multimedia Appendix 5 Customization instructions, customized avatar examples, and customized hot-air balloon examples.

Multimedia Appendix 6 Avatar for the no customization active control condition.

Multimedia Appendix 7 Detailed procedure of the 14-day app intervention.

Multimedia Appendix 8 Results of the association between anxiety, stress, and avatar identification.

Multimedia Appendix 9 CONSORT-eHEALTH checklist (version 1.6.1).

## Abbreviations

CBT: cognitive behavioral therapy
cCBT: computerized cognitive behavioral therapy
GAD-7: Generalized Anxiety Disorder-7
MDD: major depression disorder
PHQ: Patient Health Questionnaire
PSS: Perceived Stress Scale
SUS: System Usability Scale
