# Supplementary figures and images for: The Effect of Mental Health App Customization on Depressive Symptoms in College Students: Randomized Controlled Trial
Source: JMIR Ment Health. 2022 Aug 9;9(8):e39516. doi: 10.2196/39516 (PMC9399839; doi:10.2196/39516)

**Multimedia Appendix 1: Log of the Study Power Analysis**


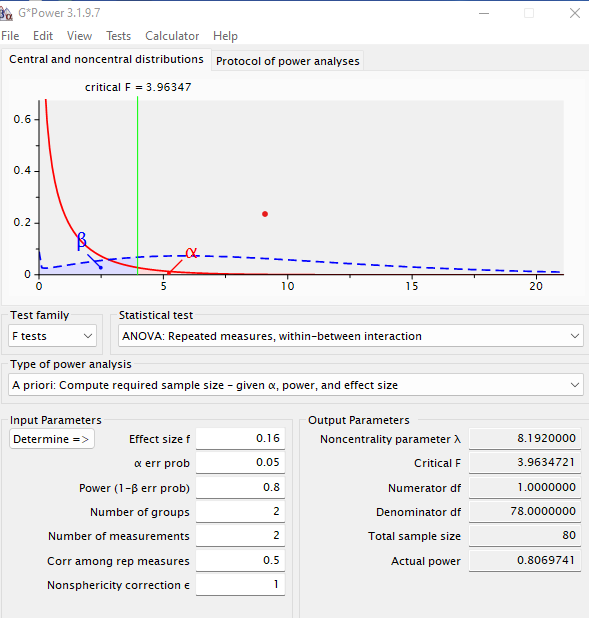

Supplement: Multimedia Appendix 1 [file mental_v9i8e39516_app1.docx]

**Multimedia Appendix 6: Avatar for the no-customization active control condition**


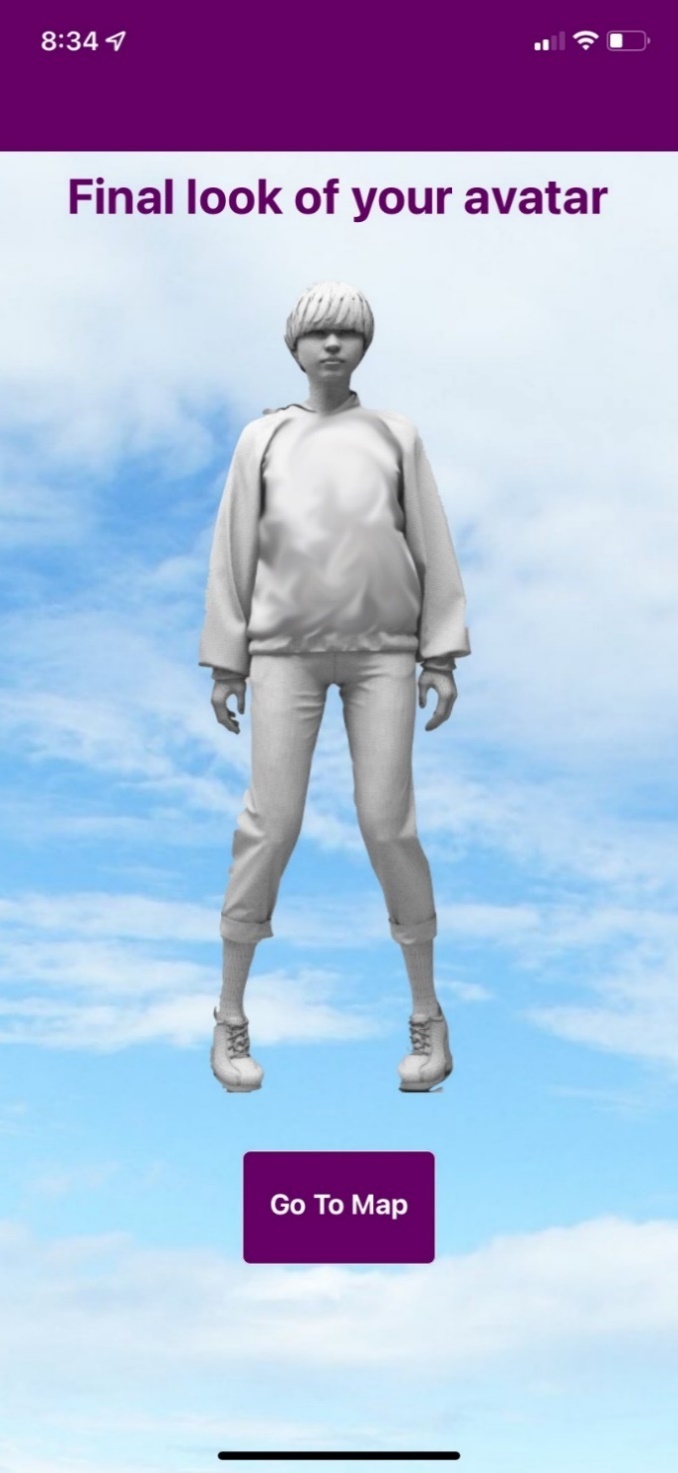

Supplement: Multimedia Appendix 6 [file mental_v9i8e39516_app6.docx]
