# Supplementary material for: The Effect of Mental Health App Customization on Depressive Symptoms in College Students: Randomized Controlled Trial
Source: JMIR Ment Health. 2022 Aug 9;9(8):e39516. doi: 10.2196/39516 (PMC9399839; doi:10.2196/39516)
Supplement: Multimedia Appendix 2 [file mental_v9i8e39516_app2.docx]

**Multimedia Appendix 2: Inclusion & Exclusion Criteria**

*Inclusion Criteria:* Inclusion criteria for participation in this study included owning an Apple iPhone or Android phone with an updated operating system, currently attending Clemson University as a student, fluency in English, having a score of five or higher on the PHQ-8, and being 18 years or older at the time the experiment begins.

*Exclusion Criteria*: Exclusion criteria excluded participants who do not have ready and bi-daily access to an Apple iPhone or Android phone with an updated operating system, does not attend Clemson University as a student, is not fluent in English, are under the age of 18, does not complete more than one CBT module in AirHeart, fails 2 or more attention checks, fails to fully complete the pre- and post-assessment, or does not consent to be a part of the study.
