# Supplementary material for: The Effect of Mental Health App Customization on Depressive Symptoms in College Students: Randomized Controlled Trial
Source: JMIR Ment Health. 2022 Aug 9;9(8):e39516. doi: 10.2196/39516 (PMC9399839; doi:10.2196/39516)
Supplement: Multimedia Appendix 3 [file mental_v9i8e39516_app3.docx]

**Multimedia Appendix 3: AirHeart App Cognitive Behavioral Therapy Module Scripts**

**Module 1: Introduction to Brief CBT & depression**

Welcome to Machu Picchu! This is the first stop along our journey to discover the secrets of the 7 wonders of the modern world. Machu Picchu was once the home of the Inca emperor Pachacuti. Just as he began his days here during his rule, so shall our adventure begin:

- What is CBT?
  - CBT stands for cognitive behavioral therapy, which is a short-term, evidence-based treatment for many problems, including depression and anxiety. It is based on science that shows that thoughts (cognitions) and behaviors (actions or choices) affect the way we feel (emotions).
    - Continue or repeat
  - CBT is regular. It works best with a consistent schedule.
    - Continue or repeat
  - CBT requires practice. It takes daily repetition to learn skills and retrain our depression-influence habits.
    - Continue or repeat
  - CBT depends on follow-through. The most important factor in whether treatment works is the amount of work you put into it.
    - Continue or repeat
- What does CBT include?
  - CBT includes:
    - Psychoeducation: information regarding depression
    - Cognitive restructuring: learning different ways to think about a problem to reduce their negative impact on your mood
    - Behavioral activation: learning how different behaviors influence emotions
    - Problem-solving skills

&

- - - Relaxation techniques
  - You will learn more about these techniques as you progress.
- Would you like to go over what is CBT or what does CBT include once more?
  - Yes ->
    - What is CBT?
    - What does CBT include?
    - Both
  - No -> continue

What is depression?

- It can mean:
  - A feeling that lasts a few minutes
  - A mood that lasts a few hours
  - A clinical condition
- Depression is:
  - How you act, like not enjoying things that were once enjoyable or interesting.
  - How your body reacts, like fatigue
  - How you feel, like sad, irritable, or unmotivated
  - How you think, like persistent negative thoughts about yourself, the world, or your future
- Everyone feels blue or sad sometimes. It’s normal to have a bad day every once in a while, shed tears after a sad movie, or feel nervous before a big day and lose sleep. These are common human experiences that are a healthy and normal part of life.
- However, when the elements listed above last for days, weeks, or longer, you may be suffering from an episode of depression
- Depression Downward Spiral
  - Regardless of the cause of the depression for you, once it starts, it often causes a downward spiral that leads to increasingly bad feelings and further negative events.
  - For example, isolating socially may cause others to stop reaching out to us and relationship may dissolve. Or our poor view of ourselves may come across to others as low confidence and people may stop respecting us or treat us poorly.
  - This cycle can take many forms: (Example then worksheet)


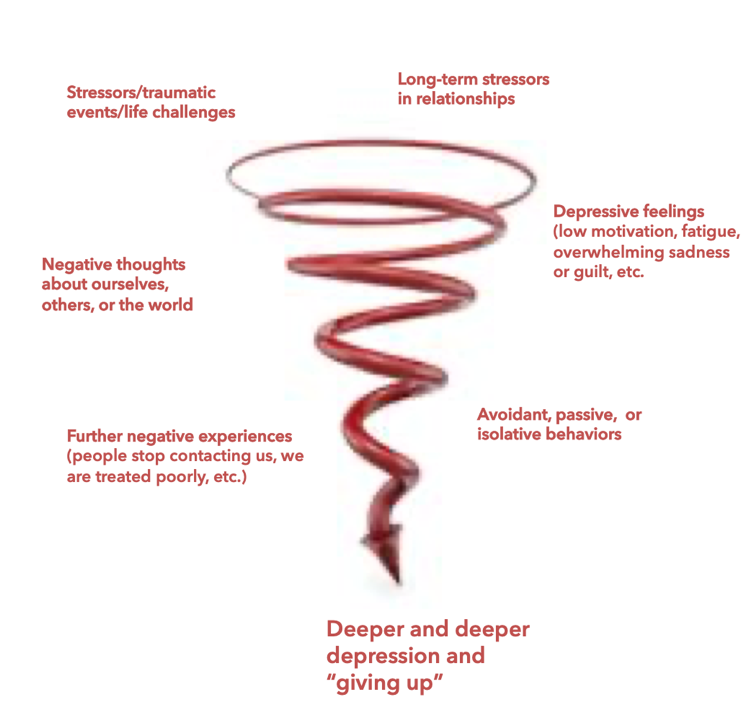


- Now that we’ve learned about CBT, depression, and identified some of your stressors, you’ve fully explored Machu Picchu. Take a day to explore, contemplate, and practice your new skills. Remember that you can re-explore Machu Picchu and practice your skills any time.

**TAKES THE USER BACK TO THE WORLD MAP**

**Module 2: Identifying Maladaptive Thoughts and Behaviors**

Welcome to Petra! This is the second stop along our journey to discover the secrets of the 7 wonders of the modern world. Petra was once the capital of an ancient civilization dating back to 7000BC. After the rise of the Byzantine empire, Petra was abandoned by most, but then rediscovered by Western influences in 1812 by a Swiss traveler. Just as Petra was rediscovered, in this module, we’re going to learn ways to discover your maladaptive thoughts and beliefs.

What are Maladaptive Thoughts

- First let’s start with what are automatic thoughts.
  - Automatic thoughts are a brief stream of thought about ourselves and others. They largely apply to specific situations and/or events and occur quickly throughout the day as we appraise ourselves, our environment, and our future. We are often unaware of these thoughts but are very familiar with the emotions that they create within us.
- A bad version of automatic thoughts is called maladaptive automatic thoughts.
  - - Maladaptive automatic thoughts are distorted reflections of a situation, which are often accepted as true.
    - Two types of these are worry and rumination:
      - Worry is related to fear that something bad might happen in the future.
      - Rumination means to chew on a thought over and over, such as repetitively reviewing a past failure.
    - For example, if Jane jumbles her words during a group presentation, a maladaptive automatic thought may present itself as “I messed up my words, no one understood me! I knew it wasn’t worth even trying during this presentation, I’m just going to fail anyway.”
- Why do I have all these negative thoughts?
  - When we are feeling depressed and anxious, our brain wants us to think about potentially dangerous things in our environment in order to keep us safe.

Identifying Maladaptive Thoughts

- Identifying maladaptive automatic thoughts is the first step in enacting change.
- Dysfunctional automatic thoughts often fall into certain categories. These are common "cognitive distortions" or thinking errors.
- Some common cognitive distortions include:
  - Filtering
    - This refers to the way a person can ignore all of the positive and good things in life to focus solely on the negative. It’s the trap of dwelling on a single [negative](https://positivepsychology.com/explanatory-styles-optimism/) aspect of a situation, even when surrounded by an abundance of good things.
  - Overgeneralization
    - This is taking a single incident or point in time and using it as the sole piece of evidence for a broad conclusion.
    - For example, someone who overgeneralizes could bomb an important job interview and instead of brushing it off as one bad experience and trying again, they conclude that they are terrible at interviewing and will never get a job offer
  - Jumping to conclusions
    - This refers to the tendency to be sure of something without any evidence at all.
    - For example, we might be convinced that someone dislikes us without having any real evidence, or we might believe that our fears will come true before we have a chance to really find out.
  - Catastrophizing
    - This involves expecting that the worst will happen or has happened, based on an incident that is nowhere near as catastrophic as it is made out to be. For example, you may make a small mistake at work and be convinced that it will ruin the project you are working on, that your boss will be furious, and that you’ll lose your job.
- When faced with an automatic thought, take a moment to assess the real emotions or beliefs behind that automatic thought.
  - For example: John was out of breath when he played in the park with his granddaughter. He automatically thought “I’m too old to play with her. I am no good to my family.” Both of these thoughts elicited feelings of defeat, hopelessness, and worthlessness.
  - Is it that John is a worthless person who cannot help his family, or perhaps John is older and not as fit?
- Identifying these automatic thoughts can further lead to revealing deeper intermediate and core beliefs.
- Here is a quick exercise to help you identify some of your own maladaptive thoughts and beliefs:
  - Please indicate a situation or situations, the automatic thought(s) which they elicited, and the emotions & mood you felt.

| 1. Situation | 1. Automatic Thought(s) | 1. Emotions & Moods |
| --- | --- | --- |
|  |  |  |

- Remember, automatic thoughts, intermediate beliefs, and core beliefs are simply ideas. Feeling them strongly does not make them real.
- Now that we’ve learned how to identify maladaptive thoughts and beliefs, you’ve fully explored Petra. Take a day to explore, contemplate, and practice your new skills. Remember that you can re-explore Petra and practice your skills any time. Your worksheet has been saved to your adventure journal.

**TAKES THE USER BACK TO THE WORLD MAP**

**Module 3: Challenging Maladaptive Thoughts and Beliefs**

Welcome to the Roman Colosseum! This is the third stop along our journey to discover the secrets of the 7 wonders of the modern world. The Roman Colosseum was constructed prior to 80 AD o host gladiator battles, hunting, and other performances. Just as gladiators used to challenge wild animals and other fighters, in this module, we will teach you how to challenge your maladaptive thoughts and beliefs.

Challenging Maladaptive Thoughts and Beliefs

- Several techniques can be used to challenge dysfunctional thoughts or beliefs, most are used in conjunction with a thought record, like your purple adventure journal.
- Challenging thoughts and beliefs allow you to use your own statements to counter dysfunctional thinking.
- Once you identify the cognitive distortions you hold (the list we learned last module), you can begin to explore how those distortions took root and why you came to believe them. When you discover a belief that is destructive or harmful, you can begin to challenge it.
- For example, if you believe that you must have a high-paying job to be a respectable person, but you’re then laid off from your high-paying job, you will begin to feel bad about yourself.
- Instead of accepting this faulty belief that leads you to think negative thoughts about yourself, you could take an opportunity to think about what really makes a person “respectable,” a belief you may not have explicitly considered before.
- Ways to challenge Maladaptive thoughts
  - Decreasing thoughts that makes us feel bad
    - Thought stopping
      - When a thought is ruining your mood:
        - Identify it:
        - Tell yourself: “This is ruining my mood:
        - Think of another thought
      - Worrying Time
        - Schedule worry time each day so you can concentrate completely on necessary thinking and leave the rest of the day worry free.
        - Worry time can be 10-30 minutes.
        - If you begin to worry about something upcoming, remind yourself that you’ve set aside a time to worry later and reorient yourself to the task at hand.
      - Consider the worst that could happen
        - Often, vague fears about what could happen make us more depressed than thinking things through and facing the worst possibilities.
        - Remember that the worst that can happen is only one of many possibilities, just because it is the worst, it is not the one most likely to happen.
  - Practice self-compassion
    - Three elements of self-compassion include kindness, being present, and common humanity.
    - Kindness: How would you talk to a friend who was talking about themselves that way? Gently, non-judgmentally, patiently.
    - Being present: Be a friend and companion to yourself. Accompany yourself in your distress.
    - Common humanity: Recognize and acknowledge that everyone struggles sometimes, even though our struggles may be different. No one is perfect.
  - Talking back to your thoughts: ABCD Method
    - Ask yourself, what are you thinking? Then try to talk back to the thought that is hurting you.
    - A is the Activating event (what happened)
    - B is the Belief or the thought you are having
      - What you tell yourself about what happened
    - C is the Consequence
      - The feeling you have because of the thought
    - D is the Dispute or talk back to the thought.
    - For example: Leo has been feeling very down, and he thinks it is because you have diabetes.
      - The Activating event is the fact that Leo has diabetes.
      - The Belief or thoughts he has about this fact are:
        - “No one can be happy if one has diabetes”
        - “Diabetes will ruin my life”
        - “I cannot stand having diabetes”
      - The Consequence of thinking these thoughts is feeling very depressed for a long time.
      - The Dispute can look something like this:
        - There are people who have diabetes who are as happy as people who don’t have diabetes.
        - Diabetes is a burden I have to deal with, but it does not necessarily ruin every minute of my life.
        - Having diabetes is unfortunate, but many human beings have diabetes
  - Take a moment to fill out this ABCD Method chart using one of your own maladaptive thoughts
- Now that we’ve learned how to challenge maladaptive thoughts and beliefs like gladiators, you’ve fully conquered the Roman Coliseum. Take a day to explore, contemplate, and practice your new skills. Remember that you can re-explore the Coliseum and practice your skills any time. Your worksheet(s) have been saved to your adventure journal.

**TAKES THE USER BACK TO THE WORLD MAP**

**Module 4: Behavioral Activation**

Welcome to Chichen Itza! This is the fourth stop along our journey to discover the secrets of the 7 wonders of the modern world. Chichen Itza was an ancient Mexican city built by the Maya people around 6^th^ century CE. This wonderous city was used as a commercial, military, political, and religious center and home to an estimated 35,000 people. Just like all the hustle and bustle of ancient life filled with plenty of activities to do, in this module, we’re going to teach you about a skill called behavioral activation which promotes an active lifestyle.

- What is Behavioral Activation?
  - Behavioral activation includes a set of procedures and techniques that help you understand how behaviors influence emotions.
  - The key here is that difficulty with mood often serves to increase avoidance of pleasant events, which help to alleviate and avoid depression.
  - Re-introducing pleasant events can serve to improve mood in many ways:
    - 1) reversing avoidance
    - 2) increasing physical activity
    - 3) increasing self-confidence
    - 4) increasing feelings of usefulness and purpose
  - Behavioral activation includes understanding the vicious cycles, monitoring our daily activities, activity scheduling, and reducing avoidance.
- Vicious Cycles
  - Do you stop doing things because you feel sad, disheartened, or depression?
  - Do you get depressed because you stop doing things?
    - The answer is both
  - More often than not, the less you do, the more depressed you feel. The more depressed you feel, the less you do. This is called a vicious cycle.
    - To break this vicious cycle, you increase those activities which make you feel better, like grabbing a cup of coffee with a friend or re-introducing a favorite hobby.
- Activity monitoring
  - This is being aware of your actions and behaviors in order to understand which behaviors help you feel better, which continue to maintain the sad, sullen moods, and which make you feel worse.
  - Make a note of whenever you start feeling sad, anxious, or any other kind of negative emotion.
  - Is there a trend? What brings about these
  - feelings?
  - Next time you feel happy or joyful, take note of what actions brought upon these feelings.
- Scheduling pleasurable activities helps prevent the downward spiral and reduces avoidance. Just telling yourself to “feel better” isn’t enough, the things that you do will change the way that you are feeling. There doesn’t have to be a special event, just the will to do the things that make you happy and feel relaxed.
- Schedule two activities this week that you want to do. These can be anything that makes you happy or that you haven’t had time to do for yourself.
- What are those 2 activities?
- 1.
- 2.
- Make sure to make a record of your emotions before and after you finish your activities. Did they improve your mood, did your mood stay the same, or was your mood dampened?
- Now that we’ve learned different behavioral activation skills, you’ve fully immersed yourself in ancient Mayan life at Chichen Itza. Take a day to explore, contemplate, and practice your new skills. Remember that you can re-explore Chichen Itza and practice your skills any time. Your worksheet(s) have been saved to your adventure journal.

**TAKES THE USER BACK TO THE WORLD MAP**

**Module 5: Problem Solving**

Welcome to Christ the Redeemer! This is the fifth stop along our journey to discover the secrets of the seven wonders of the modern world. The Christ the Redeemer statue stands on the top of Mount Corcovado in Rio de Janeiro, a populous city in Brazil. Interestingly, this statue is often struck by lightning thus leading to necessary repairs. Just as architects and engineers continue to find new and inventive ways to prevent lightning from striking the concrete image of Christ, in this module, you will learn about problem-solving.

What is problem-solving in CBT?

- This includes techniques which generally involve a process by which an individual attempts to identify effective means of coping with problems of everyday living.
- This often involves a set of steps for analyzing a problem, identifying options for coping, evaluating the options, deciding upon a plan, and developing strategies for implementing the plan.

The problem-solving technique which we’re going to teach you is called the SOLVE technique.

- This helps guide you through steps to identify and solve problems in your life. First, starting with S.
  - S: Selecting the problem
    - Think about situations when you feel distress.
    - For example, do you experience any family stress, mobility issues, managing your mood, or emotional or social problems.
    - Let’s take Georgia for example. Georgia is a 23-year-old recently diagnosed with epilepsy. She wants to find a way to reduce her physical symptoms.
  - O: Opening your mind to all solutions
    - Brainstorm all possible solution
    - Writing may be particularly helpful
    - Even ideas that seem ridiculous at first may generate realistic solutions
    - Let’s return to Georgia. What are some solutions Georgia could come up with to help her reduce her symptoms of epilepsy? Choose all that apply: *User selects the answers. If they have one that is not correct or is missing one of the correct answers, the pop-up “I think you may be missing something” or “Let’s try that one more time” appears. Once the user selects all the correct answers, then it progresses.*
      - **Talk to her doctor**
      - Keep her worries to herself
      - **Engage in a healthy lifestyle**
      - Tough out the bad times, they will make her stronger
    - Talking to her doctor or engaging a healthy lifestyle are all great solutions to Georgia’s problem.
  - L: Listing the potential pros and cons of each potential solution
    - Often, writing options, along with listing pros and cons, can be helpful in considering potential options. Writing allows additional thought, as well as a visual image of options.
    - Let’s look at the solutions Georgia came up with. First, Georgia said that she could talk to her doctor.
      - Pros:
        - Mental health professional’s opinion who is well-versed with epilepsy patients.
        - Could prescribe medication or change her current medication to further help with symptoms
      - Cons:
        - Expensive doctor’s visits and medication fees
    - The other solution was to live a healthy lifestyle.
      - Pros:
        - Exercise and eating nutritious foods will not only help the symptoms, but also make her more active and her body feel better.
      - Cons:
        - Exercise and healthy foods could be expensive
  - V: Verify the best solution
    - Examine the pros and cons of the solutions listed. You may want to rank order the solutions based on which solutions are most practical and/or desirable.
    - For Georgia, which plan do you think she should choose?
      - Talking to her doctor
      - Living a healthier lifestyle
      - Both
  - E: Enacting the Plan
    - Identify the steps needed to carry out the solution selected. Breaking down actions into small steps may help you both feel accomplished and visualize a path towards achieving your goal.
  - D: Deciding if the plan worked
    - After putting the plan into action, see if it worked. If the plan worked, great! If the plan did not work, you can always give the plan more time or choose a different option from the ideas you thought of previously.
  - You’ve seen Georgia walk through the steps of the SOLVED problem-solving strategy, now it’s your turn. Fill out each of the 6 steps to help solve one of your own problems.
- Now that you’ve learned a way to help trouble shoot any problem you may encounter, you’ve completed your exploration of the Christ the Redeemer Statue. Take a day to explore, contemplate, and practice your new skills. Remember that you can re-visit the Christ the Redeemer Statue and practice your skills any time. Your worksheet(s) have been saved to your adventure journal.

**TAKES THE USER BACK TO THE WORLD MAP**

**Module 6: Mindfulness:**

Welcome to the Taj Mahal! This is the sixth stop along our journey to discover the secrets of the seven wonders of the modern world. Widely recognized as one of the most iconic buildings in all of India, the Taj Mahal encourages tourists to come take pictures of her beautiful architecture and relaxing scenery. Just as the Taj Mahal offers a relaxing atmosphere, in this module, you will learn about different relaxation techniques.

What is mindfulness

- Mindfulness is a practice that can be helpful in calming the mind by reducing our tendency to try to control it, which often makes our emotions worse.
- Monkey Mind:
  - Does it ever feel like you are at the mercy of your thoughts? Many of us feel like our minds are constantly going from thought-to-thought like a monkey swinging from tree-to-tree.
  - This is a very common experience! The good news is: when you notice that this is happening you are taking the first step towards being mindful. You can learn to notice your distractions and come back to the present moment. We practice this over, and over, and over again. This is one way to practice mindfulness.
- Mindfulness helps us slow down to experience life as it is.
- Mindfulness Exercises
  - Slow Diaphragmatic Breathing/Deep Breathing
    - This is a developed technique that involves slowing down the breath to communicate “safety” to the brain.
    - Tips:
      - Speed of the breathing is more important than the depth of the breath.
      - Don’t use breathing exercises to “get rid of” bad emotions, use the breath to help get you through a tough situation
      - Practice!
    - Try this now as we give you instructions:
    - Steps:
      - Sit comfortably in a chair with your feet on the floor, or you may lie down
      - Fold your hands on your belly
      - Breathe in slowly and calmly. Fill up the belly with a *normal* breath. Try not to breathe in too heavily. The hands should move up when you breathe in, as if you are filling up a balloon. Avoid lifting the shoulders as you inhale; rather, breathe into the stomach
      - Breathe out slowly to the count of “5.” Try to slow down the rate of the exhale. After the exhale, hold for 2-3 seconds before inhaling again.
      - Work to continue to slow down the pace of the breath
- Next, we’re going to try progressive muscle relaxation.
  - Progressive Muscle Relaxation
    - PMR consists of learning how to tense and then relax various groups of muscles all through the body in a sequential fashion, while paying close attention to the feelings associated with both tension and relaxation.
  - Try this now as we give you instructions
    - Steps:
      - Dominant arm. Make a fist and tense biceps, notice the sensation of tightness. After a few seconds, release your grip and notice the relaxation sensation.
      - Nondominant arm. Make a fist and tense biceps, notice the sensation of tightness. After a few seconds, release your grip and notice the relaxation sensation.
      - Forehead, lower cheeks and jaw. Lift eyebrows as high as possible, bite teeth together, and pull corners of mouth tightly. Notice the sensation of tightness, and, after a few seconds, release your grip and notice the relaxation sensation
      - Neck and throat. Pull your chin down toward your chest but try to prevent it from actually touching the chest. Notice the sensation of tightness, and, after a few seconds, release your grip and notice the relaxation sensation.
      - Shoulders, chest, and upper back/abdomen. Take a deep breath and hold it. At the same time, pull the shoulder blades back and together, trying to make them touch. At the same time make the stomach hard by pressing it out, as if someone were going to hit you in the stomach. Notice the sensation of tightness, and, after a few seconds, release your grip and notice the relaxation sensation.
      - Dominant leg. Lift foot off the floor and push down on the chair with thigh. Notice the sensation of tightness, and, after a few seconds, release your grip and notice the relaxation sensation.
      - Nondominant leg. Notice the sensation of tightness, and, after a few seconds, release your grip and notice the relaxation sensation
  - You’ve now completed both mindfulness exercises!
- Now that you’ve learned several strategies for mindfulness and relaxation, you’ve fully explored the Taj Mahal. Take a day to explore, contemplate, and practice your new skills. Remember that you can re-visit the Taj Mahal and practice your skills any time. Your worksheet(s) have been saved to your adventure journal.

**TAKES THE USER BACK TO THE WORLD MAP**

**Module 7: Ending-Treatment & Maintaining Changes**

Welcome to the Great Wall of China This is the final stop along our journey to discover the secrets of the 7 wonders of the modern world. The Great Wall of China, constructed in the 7^th^ century BCE to protect against attacks from enemies, spans over 5,000 miles. Just like the Great Wall of China kept the people safe from attacks, this session is a review of all the tools you’ve gathered through these modules to ensure that you’ll be well prepared to face any challenges.

- Reviewing the past 6 modules:
  - First, we learned about CBT and depression.
    - CBT stands for cognitive behavioral therapy, which is a short-term, evidence-based treatment for many problems, including depression and anxiety. It is based on science that shows that thoughts (cognitions) and behaviors (actions or choices) affect the way we feel (emotions).
    - Depression is a negative mood, emotion, or the clinical condition which can produce a number of both physical and emotional symptoms.
  - In the 2^nd^ and 3^rd^ modules, taught us how to identify and challenge maladaptive thoughts and beliefs. By identifying cognitive distortions and automatic thoughts and beliefs, we can determine the real emotions and beliefs behind the thoughts. When challenging these thoughts and beliefs, thought stopping, ABCD Method, healthy thinking, cost/benefit analysis, and life goal analysis.
  - Activity monitoring learned in module four with Behavioral Activation and the SOLVED Technique from module five with problem-solving both provide examples of exercises to help encourage pleasant activities and reduce stress from a variety of problems.
  - Different relaxation techniques, like deep breathing, progressive muscle relaxation, and observing and describing.
  - These techniques can help to fight against negative or depressive emotions and moods.
- Take some time to revisit your favorite wonders today and re-complete some of your favorite exercises.

**CONGRATULATIONS! YOU’VE COMPLETED ALL 7 MODULES. FEEL FREE TO RETURN TO ANY OF THE WONDERS AT ANY TIME TO REFRESH YOUR SKILLS.**
