# Supplementary material for: The Effect of Mental Health App Customization on Depressive Symptoms in College Students: Randomized Controlled Trial
Source: JMIR Ment Health. 2022 Aug 9;9(8):e39516. doi: 10.2196/39516 (PMC9399839; doi:10.2196/39516)
Supplement: Multimedia Appendix 4 [file mental_v9i8e39516_app4.docx]

**Multimedia Appendix 4: AirHeart App Features**

*Feature 1: CBT Modules.* CBT modules were chosen based on their recurrent success in reducing depressive symptoms, especially in a computerized format (Watts et al., 2013; Wells et al., 2018; Wright et al., 2019). Topics for the modules focus on psychoeducation, identifying and challenging maladaptive thoughts, behavioral activation, problem-solving, and mindfulness (Ackerman 2021; Cully & Teten, 2008; Miner et al., 2016; Muñoz et al., 2000). Participants learned about the topic (e.g., “what is depression” or “what are maladaptive thoughts”) and strategies to aid in the reduction of their negative symptoms. Participants were encouraged to utilize these strategies in an exercise. Modules were completed every other day beginning with the first CBT module the day after the tutorial to provide rest and allow a day for participants to practice their newly learned skills.

*Feature 2: Login Questionnaire.* The twelve-item login questionnaire comprises of shortened versions of select surveys completed during the initial meeting, the Patient Health Questionnaire -4, Perceived Stress Scale-4, and four items from the positive affect scale in the Positive and Negative Affect Scale. Participants were able to track their pattern of mood grouped by positive and negative affect in the mood tracking line graph mentioned below. The login questionnaire was to be completed a minimum of seven times over the course of the experiment, and each login was time stamped within the app.

*Feature 3: Mood Tracking*. The response to the login questionnaire inquiring about the participant’s current emotional state, specifically their positive and negative affect, collated into a visual line graph where participants could track their progress and moods through the app. The more login questionnaires the participants completed over time, the more information the line graph was able to provide. The graph displayed two different lines, one for positive affect, positive scale of the positive and negative affect scale short version (PANAS-SF) and another for negative affect (PSS-4 & PHQ-4) differentiated by color. Means scores was shown for each day the login questionnaire was completed.

*Feature 4: Journaling*. The journaling feature offered users a place to write about their day or what they learned from the modules. When participants opened the journal, they type on white paper with size 12 Times New Roman font. Participants were encouraged to write a journal response after completing their login questionnaire before continuing to the CBT modules. The journal could also be accessed any time after the first initial journal completion of that chosen day. Participants can review old entries or add new entries. Participants were informed that researchers would not have access to journal entries, only the character count of the diction within the journal entries and a time stamp.

*Feature 5: Reminders*. Used to prompt participants and encourage adherence, reminders were utilized via the reminders or calendar app on Apple iPhones and Android phones. Setting reminders allowed for the notification to appear on the iPhone’s lock screen as a visual reminder, and audio sounds to notify participants that it is time to open the app. Participants were given options to choose when the reminder will activate every other day: either at 8 AM or 9 AM in the morning and 4 PM or 5PM, in the evening Eastern Standard Time. These times were selected for a variety of reasons: the morning options were chosen, so participants would wake up to the reminder to complete their modules. An additional nighttime reminder was chosen to allow for a portion of the day to pass, allowing experiences and emotions to formulate. Additionally, since the population in question consists of college students, classes are most commonly held from 8 AM to 5 PM. Setting the reminder later in the day allowed for students to complete their classes and potentially increased the probability that they will not be in class once the reminder chimes. The addition of a second reminder helped increase adherence by providing participants with a supplementary prompt in case the first reminder was forgotten or ignored.

*Accessing AirHeart Features.* The participants could access the seven CBT modules, the mood tracker, and the journal from the map home page. The participants could also view or edit their avatar and hot air balloon from this page. See Figure S1 for a visual.


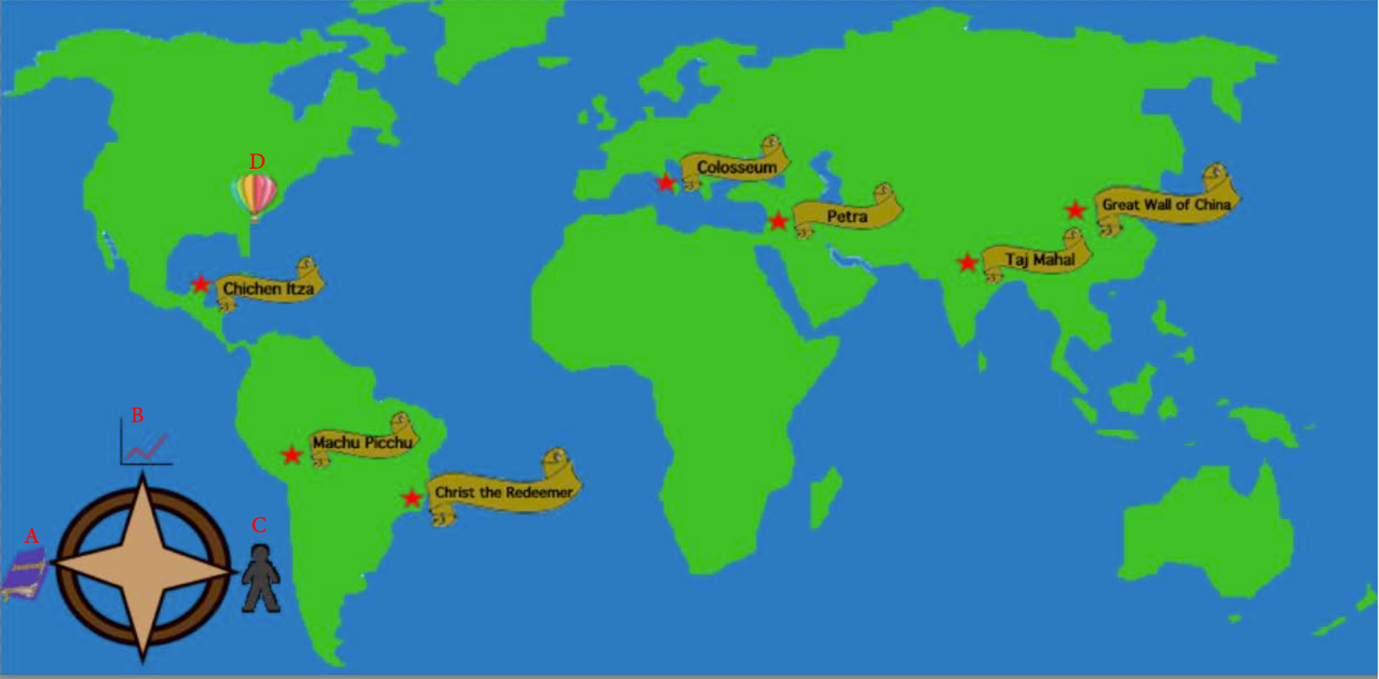


*Figure S1:* Map home page for the AirHeart app. The seven wonders of the world are indicated with red stars and labeled on the right. (A) Journaling icon which leads to the daily journal entries. (B) Mood tracking icon which leads to the line graph depicting the user’s overall and individualized scores for depression, anxiety, and stress gathered from the daily login questionnaires. (C) Avatar customization icon which allows individuals in the intervention condition to edit their avatars and individuals in the control condition to view their avatars. (D) The user’s hot air balloon icon which allows individuals in the intervention condition to edit their hot air balloon and individuals in the control condition to view their hot air balloon.
