# Supplementary material for: The Effect of Mental Health App Customization on Depressive Symptoms in College Students: Randomized Controlled Trial
Source: JMIR Ment Health. 2022 Aug 9;9(8):e39516. doi: 10.2196/39516 (PMC9399839; doi:10.2196/39516)

**Multimedia Appendix 5: Customization instructions, customized avatar examples, and customized hot air balloon examples**

**A**: The customization prompt instructing the participant to create an avatar that is the embodiment of themselves.

“Hello and welcome to AirHeart! Before we dive into the world, first you must create your character. Design this character to look like you, as it will be your virtual identity throughout the world of AirHeart. Please change the avatar’s skin, eye, and hair color, as well as clothes and accessories to match your personal style.”

**B**: Examples of customized avatars in the customization condition.


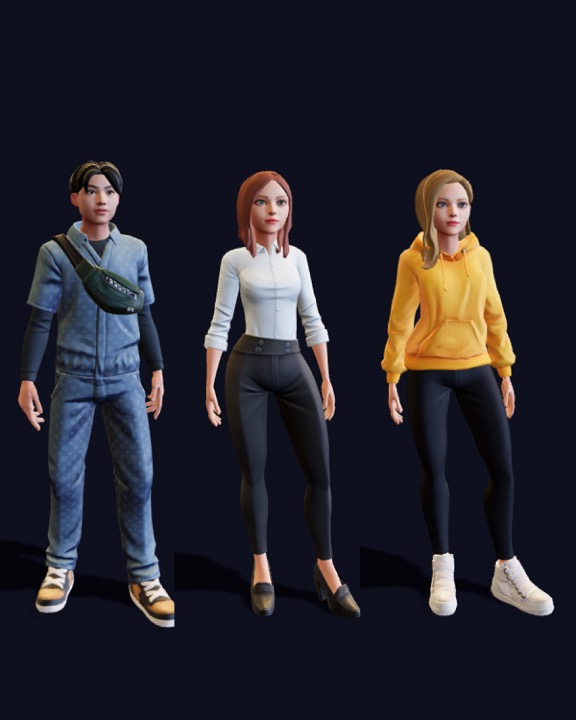


**C**: Examples of customized hot air balloons in the customization condition.

Example creation of the hot air balloons.


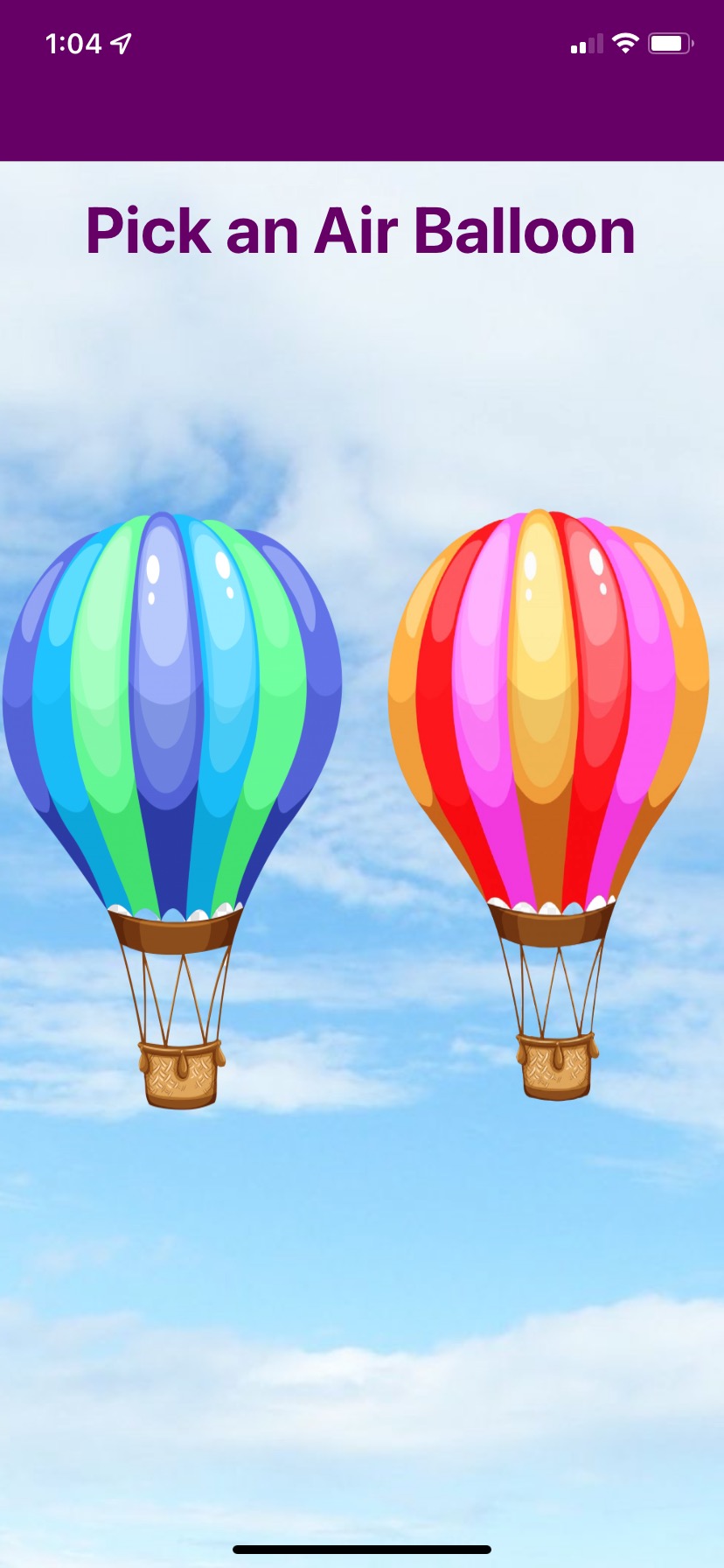

Supplement: Multimedia Appendix 5 [file mental_v9i8e39516_app5.docx]
