# Supplementary material for: The Effect of Mental Health App Customization on Depressive Symptoms in College Students: Randomized Controlled Trial
Source: JMIR Ment Health. 2022 Aug 9;9(8):e39516. doi: 10.2196/39516 (PMC9399839; doi:10.2196/39516)
Supplement: Multimedia Appendix 7 [file mental_v9i8e39516_app7.docx]

**Multimedia Appendix 7:** **Detailed procedure of the 14-day app intervention**

Intervention Period

The intervention period lasted for 14-days, after which a post-intervention survey was sent to all participants. A typical day of app usage would commence by opening the AirHeart app which followed a similar process to the one seen in the tutorial beginning with the twelve login questions regarding their mood. Once completed, the participants were prompted to complete a journal entry. After completing their login journal entry, the participant then navigated to the main map. There, they were shown their hot air balloon on the main map on the starred location which was the next wonder to complete the newest CBT module. Participants could return to their journal to make a note of their new skill set, view their progress at the mood tracker, revisit any previous wonder to rediscover a module, or, in the intervention group, edit their avatar. Whenever the participant was ready, they would complete the new module. After completing the module, participants had the same three or four options as prior to completing the new module. After completing any variety of these options, once the user has finished, they closed the app.
