# Supplementary material for: The Effect of Mental Health App Customization on Depressive Symptoms in College Students: Randomized Controlled Trial
Source: JMIR Ment Health. 2022 Aug 9;9(8):e39516. doi: 10.2196/39516 (PMC9399839; doi:10.2196/39516)
Supplement: Multimedia Appendix 8 [file mental_v9i8e39516_app8.docx]

**Multimedia Appendix 8: Results of the association between anxiety, stress, and avatar identification**

**Table S1.** Correlations between the GAD-7 anxiety & PSS-10 stress scores after 14 days and the Avatar Identification Questionnaire

|  | **Anxiety (GAD-7)** | | **Stress (PSS-10)** | |
| --- | --- | --- | --- | --- |
| **Variable** | **r** | **Sig. (p-value)** | **r** | **Sig. (p- value)** |
| Identified with Avatar | 0.068 | 0.700 | 0.159 | 0.362 |
| Connection with Avatar | 0.154 | 0.378 | 0.256 | 0.138 |
| Avatar was Not Like Me | -0.075 | 0.666 | -0.028 | 0.873 |
| Avatar is More Accomplished | 0.042 | 0.810 | -0.067 | 0.701 |
| I Like My Avatar | 0.065 | 0.711 | 0.348^*^ | 0.040 |
| Avatar Made AirHeart More Enjoyable | -0.040 | 0.820 | 0.276 | 0.109 |
| Avatar Made Me Want to Use AirHeart | 0.082 | 0.639 | 0.169 | 0.331 |
| Avatar Helped During Modules | -0.102 | 0.559 | 0.141 | 0.418 |

*Note*. * Correlation is significant at the 0.05 level (2-tailed). r value reflects the Pearson correlation.
